# Conventional and biodegradable microplastics elicit contrasting taxon-level responses in rhizosphere microbiomes of maize and strawberry

**DOI:** 10.1093/femsec/fiag040

**Published:** 2026-04-22

**Authors:** Aileen Jung, Ryan Bartnick, Daniel Churchill Thomas, Eva Lehndorff, Tillmann Lueders

**Affiliations:** University of Bayreuth, Ecological Microbiology, Bayreuth Center of Ecology and Environmental Research (BayCEER), Dr.-Hans-Frisch-Str. 1-3, 95448 Bayreuth, Germany; Marine Biological Laboratory, Ecosystems Center and J. Bay Paul Center for Comparative Molecular Biology and Evolution, Woods Hole, MA 02543, United States; University of Bayreuth, Soil Ecology, Bayreuth Center of Ecology and Environmental Research (BayCEER), Dr.-Hans-Frisch-Str. 1-3, 95448 Bayreuth, Germany; University of Bayreuth, Ecological Microbiology, Bayreuth Center of Ecology and Environmental Research (BayCEER), Dr.-Hans-Frisch-Str. 1-3, 95448 Bayreuth, Germany; University of Bayreuth, Soil Ecology, Bayreuth Center of Ecology and Environmental Research (BayCEER), Dr.-Hans-Frisch-Str. 1-3, 95448 Bayreuth, Germany; University of Bayreuth, Ecological Microbiology, Bayreuth Center of Ecology and Environmental Research (BayCEER), Dr.-Hans-Frisch-Str. 1-3, 95448 Bayreuth, Germany

**Keywords:** soil contaminants, plastisphere, agricultural soils, *Zea mays*, *Fragaria × ananassa*, metabarcoding

## Abstract

Microplastics (MP) are relevant contaminants in agroecosystems, influencing soil nutrient dynamics and soil–plant–microbial interactions. As agriculture shifts from conventional to biodegradable plastics, their impacts on different crop rhizosphere microbiomes considering both total (DNA-derived) and transcriptionally active (rRNA-derived) communities have not been clearly elaborated. We hypothesized that microbiome impacts would be distinct across different plants and polymer types. Maize and strawberry plants were cultivated with 1% MP by soil weight, including two conventional polymers (low-density polyethylene—LDPE and polyethylene terephthalate—PET) and one biodegradable polymer (poly(butylene adipate-co-terephthalate)—PBAT). Strawberry plants increased biomass across all MP treatments, accompanied by greater soil nitrate depletion. MP-induced impacts on soil prokaryotic communities were mostly additive to plant effects, as determined by 16S rRNA amplicon sequence profiling. PBAT stimulated *Cupriavidus* spp. and members of *Saccharimonadales*, suggesting a selection of potential polymer-degraders and microbial interactions, independent of plant species and root proximity. In contrast, conventional MPs induced a less selective response with compositional shifts across a greater number of taxa. MP-induced changes were more apparent in rRNA- than DNA-derived profiles, suggesting a profound response of putative active taxa. Together, we demonstrate that plant species and MP type jointly modulate rhizosphere microbial community response to MP pollution, with direct implications for soil biogeochemistry, rhizosphere functioning, and crop performance.

## Teaser

Microplastic effects on rhizosphere microbiomes depend on both plant species and polymer type, with biodegradable plastics selectively stimulating potential degraders and conventional plastics causing broader community shifts, particularly among transcriptionally active taxa.

## Introduction

Soil microorganisms are a pivotal component of agroecosystems, sustaining soil functionality and promoting crop productivity. In recent decades, awareness of the impacts of anthropogenic pollution on agroecosystems has increased, with microplastic (MP) pollution raising specific concerns (Rillig et al. 2017). The accumulation of MP, often defined as synthetic polymer particles ranging in size from 1 to 1000 µm (Hartmann et al. 2019), can result in a loss of soil functions and fertility by affecting soil microbiology and nutrient cycling (Bouaicha et al. 2022). Pollution levels in soil show substantial variability and are influenced by land use, geographic location, litter control, and also challenges in detection (Bläsing and Amelung 2018, Büks and Kaupenjohann 2020, Wrigley et al. 2024). In arable soils, MP can originate from many sources, such as littering and road runoff, atmospheric deposition, or direct introduction via agricultural practices (Steinmetz et al. 2016, Kernchen et al. 2022, Weber et al. 2022, Braun et al. 2023, Bhattacharjee et al. 2025). Given their varied origins, MPs are considered a highly heterogeneous suite of contaminants, varying widely in size, shape, composition, and chemical properties (Hartmann et al. 2019, Koelmans et al. 2022). Even more so, their impact within soils depends on a plethora of factors, including MP-derived characteristics, edaphic, and plant-mediated factors (Zantis et al. 2023b, Lozano et al. 2021, Neubert and Brüggemann 2025). Together, these highlight the continued need for targeted studies on MP impacts on plants, soil functions, and soil microbial communities to predict environmental consequences.

MPs can alter soil physico-chemical properties in multiple ways: blocking or enlarging soil pores, reducing or increasing aggregate stability, modifying water-holding capacity, and affecting nutrient availability, depending on polymer type, shape, concentration, and soil type (Souza Machado et al. 2018, Yu et al. 2020, Nuñez et al. 2025). Besides indirect effects, MP can also directly select for specific microbiota, distinct in diversity and functional potential (Hu et al. 2023, Song et al. 2023). Microbial shifts, resulting from differences in polymer degradability, could widely impact soil carbon and nutrient cycling (Yaghoubi Khanghahi et al. 2026). In particular, biodegradable plastics have been shown to promote soil respiration and to stimulate N- and P-acquiring enzyme activities compared to more recalcitrant conventional plastics, and potentially select for microbes capable of degrading these polymers (Rauscher et al. 2023, Rüthi et al. 2023, Li et al. 2025). In contrast, the impacts of conventional, persistent plastic types on soil microbial communities and functions are comparatively less defined and more variable (Yaghoubi Khanghahi et al. 2026). As the transition from conventional to biodegradable plastics in agriculture is currently propagated, it is important to assess potential ecosystem-level impacts.

Plants selectively recruit their root-associated microbiomes, which differ in composition and activity from that of unplanted soils (Berg and Smalla 2009). The rhizosphere is continuously supplied with labile carbon and energy substrates, creating an environment with increased microbial activity, many of which are beneficial to the plant host (Hartmann et al. 2009). As the distance from the root increases, the influence of root-derived resources diminishes, leading to lower microbial activities and population densities (Kuzyakov and Razavi 2019). This spatial heterogeneity in microbial activities and resource availability may modulate the fate and impacts of MP, which has rarely been addressed in respective exposure experiments. MPs can also influence plant performance as summarized by Zantis et al. (2023a), which potentially further shape root-associated microbial communities.

Despite a growing body of literature on the effects of MP on soil microbiota, most studies to date have relied on 16S rRNA gene amplicon sequencing. While SSU rRNA gene amplicon analysis typically captures DNA from a total microbiome, it also recovers DNA from inactive, dormant, or extracellular sources (Pietramellara et al. 2009, Carini et al. 2016). In contrast, only a few studies have queried the impacts of MP on transcriptionally active microbial communities by targeting 16S rRNA transcripts (Cazaudehore et al. 2023, Garrison et al. 2024, Golwala et al. 2024). These profiles are derived from reverse transcription of ribosomal RNA into cDNA, followed by 16S rRNA amplicon sequencing. These profiles can emphasize taxa with elevated ribosomal RNA levels and can be interpreted as populations with elevated protein synthesis potential, possibly reflecting active physiological states or responses, including metabolic or stress response, or growth (Blazewicz et al. 2013). We posit that jointly assessing 16S rRNA gene- and transcript-derived community profiles may provide more comprehensive insights into microbial responses to MP pollution by distinguishing total detectable taxa from those with a potential physiological impact.

This study provides a comprehensive perspective on the impacts of biodegradable and conventional MP on soil prokaryotic communities for two contrasting crop plants, addressing total (DNA-based) and transcriptionally active (rRNA-based) microbiomes at varying distances from the root, alongside selected soil nutrients (total nitrogen, nitrate, and plant-available phosphorus). Specifically, we hypothesize that: (i) the effects of different MP types on plant biomass and soil nutrient content vary between plant species; (ii) soil prokaryotic communities are shaped by plants, with additional effects induced by MP that vary between plant species and soil compartment; (iii) conventional and biodegradable MP induce distinct polymer-specific changes in microbial communities; and finally (iv), that rRNA-based microbiome profiling reveals more pronounced compositional changes than a DNA-based approach.

To achieve the objectives of this study, we cultivated maize (*Zea mays*) and strawberry (*Fragaria* × *ananassa*) plants in a silty loam artificially contaminated with pristine MP. Both crops are typically exposed to elevated plastic levels under common agricultural practices. Strawberry plants, as a horticultural crop, are typically cultivated with plastic mulching films (Goldberger, DeVetter and Dentzman 2019), while maize, a globally important cereal, is often grown from plastic-coated seeds or on fields receiving sewage residues (Bertling, Zimmermann and Rödig 2020 , FAO 2021). This increased potential of MP accumulation makes them particularly relevant for studying MP effects and their implications for nutrient cycling and crop productivity. Moreover, their contrasting growth characteristics, nutrient demands, and root architecture enable a comparative assessment of rhizosphere MP effects and plant-specific modulation of these impacts. We included two conventional (low-density polyethylene—LDPE and polyethylene terephthalate—PET) and one biodegradable (poly(butylene adipate-co-terephthalate)—PBAT) thermoplastic, all of which are widely introduced into arable soils via common agricultural practices (FAO 2021).

## Materials and methods

### MP preparation

MP fragments in a size range of 75–400 µm were generated from a single cryogenic (dry ice and liquid nitrogen) grinding cycle of LDPE (Lupolen 1800 P; LyondellBasell, Germany), PET (CleanPET WF; Veolia, Germany), and PBAT (M-VERA® B5026; BIO-FED, Germany) with a ZM 200 ultra-centrifugal mill (Retsch, Germany). To obtain the targeted size range, materials were sieved with an air-jet sieving machine (e200 LS, Hosokawa Alpine, Germany), and the resulting 75–200 µm and 200–400 µm size fractions were pooled in a 1:6 weight ratio (w/w), which was necessary to provide sufficient material for our microcosms, given the limited yield of the smaller fraction.

### Experimental setup and sampling

The impact of MP on bulk and rhizosphere prokaryotic communities was investigated in a greenhouse experiment using a two-factorial design (Fig. S1A). Top soil of a silty loam (sampled 0–30 cm; see Table S1 for basic soil properties) was collected, sieved (2 mm), dried (40°C), and homogeneously mixed with 1% (w/w) pristine MPs of either LDPE, PET, or PBAT. While MP concentrations reported from field samples can vary over several orders of magnitude, average concentrations detected in agricultural soils are usually in the ppm range, with soil pollutions in the % range are rarely reported (Büks and Kaupenjohann 2020). However, many experimental studies have used concentrations in this range (Qi et al. 2020, Sun et al. 2022), which likely triggered a more tangible and mechanistically informative response of exposed soil microbiota while overcoming dilution effects. Control treatments consisted of soil without MP amendments (no MP). Maize seeds (*Z. mays*, cultivar Benedictio KWS, ID: 18V-7004, untreated seed; KWS Saat, Germany) were washed with deionized and demineralized water, surface-sterilized with 10% H_2_O_2_ for 8 min, rinsed with water, and germinated at 30°C in Petri dishes on filter paper. Seedlings were transferred to the rhizoboxes once both radicle (∼1.2 cm length) and coleoptile had emerged (rhizobox design shown in Fig. S1B). Bare-rooted and cold-stored Frigo strawberry plants (*Fragaria* × *ananassa*, cultivar Honeoye, Gartenversandhaus Chrestensen, Germany) were directly planted in rhizoboxes. Greenhouse temperature during growth was 21.5 ± 2.9°C, and air humidity was 55.6 ± 8.3%. Soils in the rhizoboxes had an initial gravimetric water-holding capacity of 60%, which was approximately maintained throughout the incubation period. Rhizoboxes were randomized within each plant group and rotated regularly among all stands throughout the incubation period. The experimental period concluded when both plant species reached the spatial limitations of the rhizoboxes, after 80 days for maize and 105 days for strawberries. By then, maize plants had reached the reproductive growth stage, and strawberries had undergone one fruit development cycle. Our experimental design comprised planted rhizoboxes and unplanted controls. Unplanted microcosms had three replicates per treatment (each replicate being an individual rhizobox), maize-planted microcosms had five, and strawberry-planted microcosms had seven replicates for plastic treatments and five for the no MP controls. We sampled three soil compartments at varying distances from plant roots to examine the differential impacts of MP on soil and rhizosphere prokaryotic communities. Soil from planted rhizoboxes, hereafter referred to as rhizosphere soil, included *root-attached soil*, defined as soil directly adhering to the root surface, and *root-distant soil*, defined as soil not in direct contact with roots but still influenced by the plant. In addition, *bulk soil* was collected from unplanted rhizoboxes. Soil sampling was conducted destructively at the end of the experimental period. Bulk and root-distant soil was collected from the entire rhizobox after discarding the top 4 cm of soil, homogenized, and a composite sample was taken to represent the full depth of rooted soil. Root-attached soil was collected by manually shaking the entire plant root system for 5 min, ensuring only tightly attached soil remained on the root. This soil was washed off with sterile tap water in an overhead shaker for 10 min. Soils for nucleic acid extraction were flash-frozen in liquid nitrogen immediately after sampling and stored at −80°C until further analysis. Aboveground and belowground plant biomass was harvested, dried at 65°C, and weighed to determine dry biomass.

### Soil carbon and nutrient analyses

After the experiment, bulk and root-distant soils were sieved (2 mm) and homogenized for carbon (C) and nutrient analysis; root-attached soil was not analysed due to limited sample size. For total C and nitrogen (N) measurements, soils were air-dried at 40°C, ball-milled, and analysed in a CN elemental analyser (Flash EA 1112, ThermoQuest, Italy). Soil nitrate and available phosphorus (P) were determined from fresh soil samples, and dry weight was determined at 105°C. Soil available nitrate was determined using a standard extraction filtration method with 1 M KCl (Mulvaney 1996), and concentrations were measured via flow injection analysis (FIA-LAB, MLE Dresden, Germany). Readily bioavailable and moderately labile (plant-available) P were determined in the first two fractions of the Hedly extraction method (Hedley et al. 1982, Tiessen and Moir 2007). Using anion-exchange strips in soil solutions, the first fraction of the extraction was collected, representing soluble, readily bioavailable P (P_sol_), primarily as inorganic phosphate (PO_4_^3-^), and labile organic P (0.5 M NaHCO_3_). The second fraction (0.5 M HCl) represented moderately labile, plant-available Fe- and Al-bound P (P_bound_). The NaHCO_3_-extractable P_sol_ from the first step was considered directly available to microbes and plants, while the HCl-extractable P can become available over time through mineral dissolution (Hedley et al. 1982, Tiessen and Moir 2007). Total plant-available P (P_available_) is the sum of both extracted fractions. All P concentrations were analyzed via ICP-OES (5800, Agilent Technologies, Germany).

### Nucleic acid extraction, PCR library preparation, and amplicon sequencing

DNA and RNA were coextracted using a phenol–chloroform extraction protocol from 500 ± 100 mg wet soil, modified after Lueders et al. (2004) and as described in Rauscher et al. (2023). After extraction, nucleic acids were purified from co-occurring contaminants (Giguere et al. 2021) using the OneStep PCR Inhibitor Removal Kit according to the manufacturer’s manual (catalog number D6030, Zymo Research, USA). RNA was further purified by DNA digestion using the TURBO DNA-free™ Kit (catalog number AM1907, Invitrogen, USA) as described in the manual. Successful gross DNA digestion was verified via 1.5% agarose gel electrophoresis. cDNA synthesis was done with the LunaScript® RT SuperMix Kit (catalog number E3010, New England Biolabs, USA). Further, DNase treatment efficiency was evaluated by 16S rRNA gene qPCRs on no-reverse transcription (no-RT) controls, in which reverse transcriptase was omitted to detect residual DNA. qPCRs were performed according to Varsadiya et al. (2025) using Takyon NoRox SYBR MasterMix Blue (Eurogentec, Belgium) with primers Ba519f / Ba907r (5′-CAGCMGCCGCGGTAATA-3′, 5′-CCGTCAATTCCTTTGAGTTT-3′). The highest observed signal in no-RT controls corresponded to ≤ 8.7 gene copies per reaction, representing max. 0.17% of the corresponding RT-qPCR signal, as determined from an external standard curve (efficiency = 72%–91%, *R*² = 0.99). An extraction blank and several no-template controls were included, with background 16S rRNA gene amplification in these controls did not exceed 9.8 gene copies per reaction. Before 16S rRNA amplicon preparation, DNA and RNA quantity and integrity were assessed by agarose gel electrophoresis and spectrophotometric analysis (NanoDrop, Thermo Fisher Scientific, USA). V4 regions of prokaryotic 16S rRNA genes were targeted using the universal primer pair 515F (Parada et al. (2016), 5′-GTGYCAGCMGCCGCGGTAA-3′) and 806r (Apprill et al. (2015), 5′-GGACTACNVGGTWTCTAAT-3′), including a phasing spacer of different length (0–2 bases) between the Illumina adapter and target region on the forward primer. PCR amplifications were conducted using the NEBNext® High-Fidelity 2X PCR Master Mix (catalog number M0541; New England Biolabs) according to the manufacturer’s instructions, with the addition of 4 µg bovine serum albumin (20 μg/μl; Roche, Switzerland) per 25 µl reaction. Thermocycling was performed as follows: an initial denaturation at 98°C for 30 s, followed by 25 cycles of denaturation at 98°C for 10 s, annealing at 51°C for 30 s, and extension at 72°C for 30 s, lastly a final extension at 72°C for 2 min. Each sample was amplified in duplicates and pooled before further processing. PCR product quality and concentration were evaluated using capillary electrophoresis on a Fragment Analyzer (Agilent Technologies, USA). Amplicons were purified with the NucleoMag 96 PCR purification kit (catalog number 744100, Macherey-Nagel, Germany). Indexing was performed in a subsequent PCR using the Nextera XT Index Kit v2 (catalog number FC-131–2001, Illumina, USA). Sample libraries were pooled at equimolar amounts, purified, and the amplicons were further size-selected using Pippin Prep Auto-Gel Purification (SAGE Science, USA). Sequencing was carried out on an iSeq™ 100-System (Illumina) in a custom 300 bp single-end format. For both maize and strawberry plants, DNA- and rRNA-derived amplicons were sequenced alongside bulk soil from unplanted rhizoboxes, with DNA and rRNA analysed in separate runs. We refer to these as plant DNA and rRNA libraries (e.g. maize DNA and maize rRNA). Raw reads were demultiplexed, Illumina adapters and primer sequences were trimmed before data deposition in the European Nucleotide Archive under accession number PRJEB95106.

### Downstream amplicon processing

Raw read processing and community analyses were carried out in R (version 4.5.0) for each library separately due to differences in quality metrics. The DADA2 pipeline version 1.36.0 (Callahan et al. 2016) was used for raw read processing. Reads were quality-filtered and truncated to 283 bp. Default DADA2 parameters were used, except for the maxEE parameter (maximum expected error), which was set to 3, and the pooling approach was used for sample interference. Taxonomic assignment of amplicon sequence variants (ASVs) to the Silva database version 138.1 (Quast et al. 2013) was done by using a trained dataset (https://doi.org/10.5281/zenodo.4587955). Negative controls were included during the entire molecular workflow to account for contaminants, which were identified using the decontam package (version 1.28.0; Davis et al. 2018) based on the prevalence filtering with a default threshold of 0.1 ASVs classified as chloroplasts and mitochondria (0.06%–0.81%), or unassigned ASVs at the kingdom or phylum level were removed before downstream analysis. After raw read processing, the libraries contained between 874 616 and 1 985 401 recovered reads, with 1141–3128 unique ASVs, a minimum of 1826, and a maximum of 13 419 reads per sample.

Alpha diversity metrics (observed ASVs, Shannon entropy, and Pielou’s evenness) were calculated from square root-transformed ASV counts with the functions provided in the vegan package (version 2.6.10; Oksanen et al. 2001). To account for differences in sequencing depth, 1000 bootstrap rarefactions (“rrarefy” function, vegan package, without replacement, and minimum sample sequencing depth) were performed and subsequently averaged. Averaged Bray–Curtis dissimilarities were computed from 1000 subsampling iterations of square root-transformed ASV counts, standardized to the sample with the lowest read depth using the “avgdist” (vegan) function. Nonmetric multidimensional scaling (NMDS) was then performed on the resulting Bray–Curtis distance matrices using the “metaMDS” function (vegan), with 20 minimum random starting points.

### Statistical analyses

Statistical analyses were performed using R version 4.5.1, with the following packages: car (version 3.1–3; Fox and Weisberg 2019), lme4 (version 1.1–37; Bates et al. 2015), stats (version 4.5.1, base), emmeans (version 1.11.1; Lenth 2023), effectsize (version 1.0.1; Ben-Shachar et al. 2020), rstatix (version 0.7.2; Kassambara 2019), FSA (version 0.10.0; Ogle et al. 2015), rcompanion (version 2.5.0; Mangiafico 2016), vegan (version 2.6.10; Oksanen et al. 2001), and ANCOMBC (version 2.10.0; Lin and Peddada 2024). Plots were created using ggplot (Wickham 2016) and ComplexHeatmap (Gu et al. 2016) packages.

#### Soil carbon and nutrients

For each response variable, residuals from the fitted model were confirmed for normality using the Shapiro–Wilk test (“shapiro.test”, stats package) and for homogeneity of variance using Levene’s test (“leveneTest”, car package). A one-way analysis of variance (ANOVA) was performed to evaluate each treatment group (factor: plant species × MP type). *Post hoc* comparisons were conducted using Tukey’s HSD, and significance letters (compact letter displays) indicate differences among treatments within the same plant species. Statistical outliers were identified and removed. Statistical significance was defined as *P* < .05.

#### Plant biomass

Differences in aboveground (sum of stem, leaves, and fruits dry weight), belowground (roots), and total biomass (sum of aboveground and belowground biomass) were assessed as a function of MP treatments by fitting three separate simple linear regression models (“lm” function, lme4 package). For each model, assumptions were evaluated by extracting the residuals from the fitted models. Normality was verified using histograms, Q-Q plots, and the Shapiro–Wilk test (“shapiro.test”, stats package). Variance homogeneity was tested using Levene’s test (“leveneTest”, car package). We performed a Type II ANOVA using the “Anova()” function (car) to test the statistical significance of models with MP treatments as predictors of biomass relative to null and intercept-only models, and estimated the effect size (“omega_squared”, effectsize package). Using our linear models, *post hoc* contrasts were conducted among the estimated marginal means of biomass for MP treatments and the no MP-control (“emmeans”, emmeans package) with the Holm correction for multiple testing.

#### Community diversity

Statistical differences in Shannon entropy across the explanatory variables, soil compartment, and MP type were assessed using the Kruskal–Wallis test (“kruskal_test’’, rstatix package), followed by Dunn’s rank sum *post hoc* test for pairwise comparisons (“dunnTest”, FSA package), as parametric assumptions were not met. *P*-value adjustment for multiple comparisons was done with a Benjamini–Hochberg correction. Significant differences between groups are indicated using compact letter displays (“cldList”, rcompanion package).

The impact of soil compartment and MP type on the community structure was assessed using permutational multivariate analysis of variance (PERMANOVA, “adonis2” function, vegan package) based on Bray–Curtis dissimilarities, with 1000 permutations to test for statistical significance. Within-group dispersion was assessed with the “betadisper” function (vegan), followed by ANOVA (“anova”, stats package) to test for differences in variation among groups. For pairwise comparisons of soil compartments, the libraries were subsetted according to the relevant factor levels, and significance was assessed using the “adonis2” function (vegan), with Benjamini–Hochberg correction applied to control the false discovery rate.

#### Distance-based redundancy analysis

The co-occurring impacts of MP, plant species, soil carbon and nutrients shaping the prokaryotic community composition were assessed by a distance-based redundancy analysis (db-RDA). Square-root-transformed Bray–Curtis distance matrices for bulk and root-distant soil communities were used as the response matrix. Root-attached soils were excluded as soil nutrient data were not available for these samples. Before analysis, all continuous variables were standardized (mean-centered) and scaled to unit variance using the function “scale” to ensure equal weighting across variables. The gradient length of the distance matrices was assessed by detrended correspondence analysis (“decorana”, vegan) to justify the use of linear constrained ordinations. Initially, a full db-RDA with all explanatory variables (soil compartment, MP type, soil C, soil N, soil nitrate, P_sol_, P_bound_, and total biomass) was built (“dbrda”, vegan). Full model significance compared to a null model was assessed using permutation-based analysis of variance (“anova.cca”, vegan) with 999 permutations. Collinearity between explanatory variables was evaluated using variance inflation factors (“vif.cca”). All explanatory variables of the final db-RDA model were treated as fixed effects with categorical variables as factors (MP type, soil compartment). Soil nutrients (N, nitrate, P_sol_, and P_bound_) and total plant biomass were highly collinear and subjected to dimensionality reduction via principal components analysis (“rda”). Variance among these variables was largely explained by the first two PC axes (71%–79% and 14%–15%), so these two PC axes were then used as composite variables in our explanatory matrix for the downstream db-RDA. The significance of the explanatory variables was evaluated using permutation-based ANOVA (“anova.cca”, by = “terms”, 999 permutations). We initially included interaction terms between MP type and soil compartment in our models, but due to nonsignificance, we retained the additive model with main effects only.

#### Differential abundance analysis

Abundance changes in individual taxa were assessed with the “ancombc2” function (ANCOMBC package). Here, we used absolute, nonrarefied ASV counts, aggregated to genus level or lowest resolved taxonomic rank. Using default settings, linear models were specified as ∼MP type × soil compartment. Dunnett-type pairwise comparisons were used to test each MP treatment against the unamended control, with Benjamini–Hochberg correction applied to adjust *P*-values for multiple testing. Taxa were considered differentially abundant if they showed an adjusted *P*-value < .05 and passed the built-in sensitivity analyses to control for false positives.

## Results

### Impacts of MPs on soil nutrient availability and plant biomass yield

**Figure 1:**
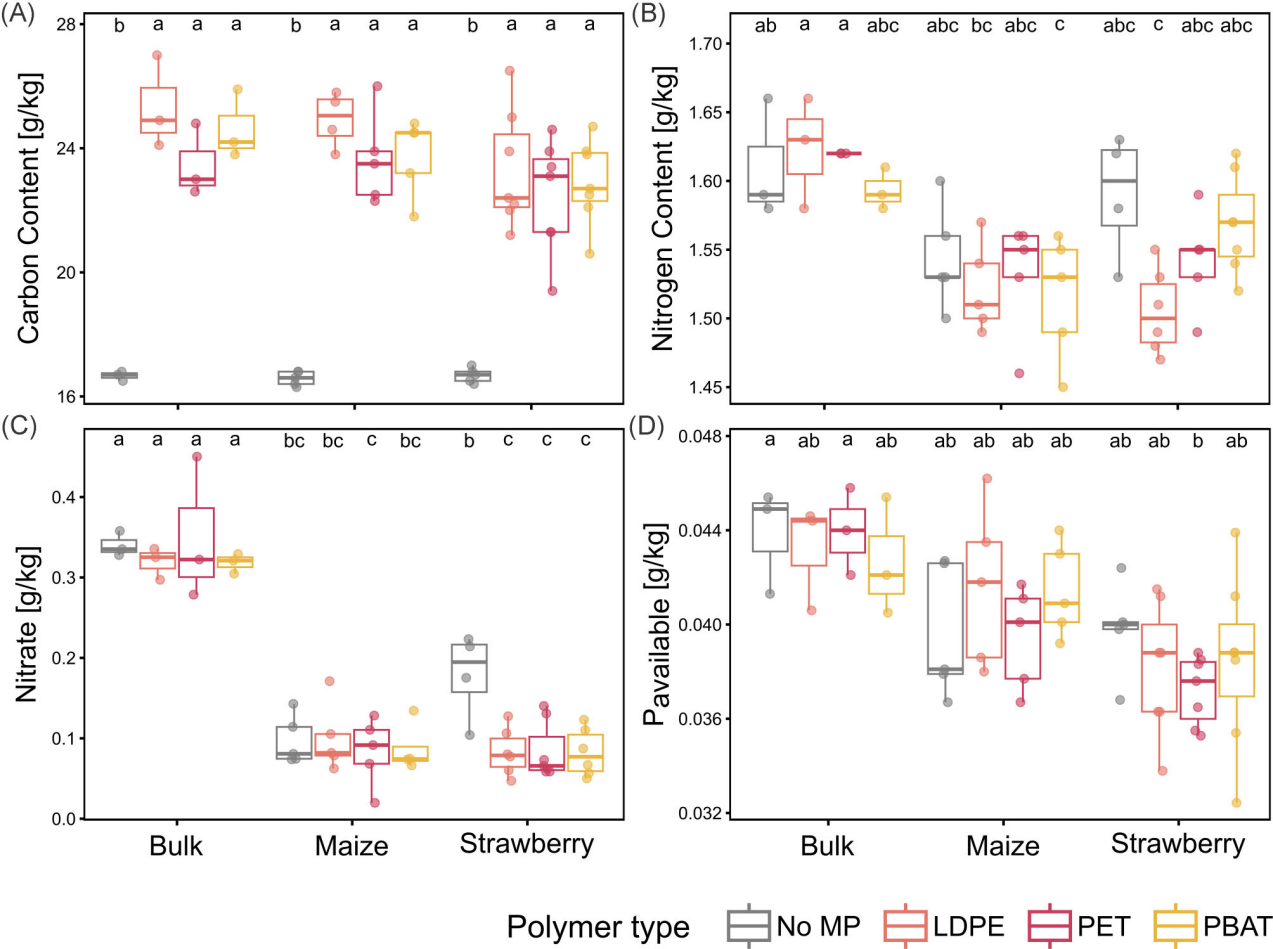
Effects of MP amendments (No MP, LDPE, PET, and PBAT) on (A) total carbon, (B) total nitrogen, (C) available nitrate, and (D) plant-available phosphorus in unplanted (bulk) and planted (maize and strawberry) soils. Boxplots show median, interquartile range, and outliers; different group letters indicate statistically significant differences among treatments within the same plant species (Tukey’s HSD, *P* < .05; same letter = no significant difference).

Total C in soil increased with MP inputs compared to controls, with total amounts increasing proportionally to the amended C in MP (Fig. 1). Plant presence or species had no significant effects on soil total C. Soil total N was reduced with plant growth, especially in maize, but significant differences between MP types were not observed. Soil nitrate decreased for planted rhizoboxes, with strawberry MP treatments showing a stronger reduction (0.08 ± 0.03 g/kg) compared to controls (0.18 ± 0.05 g/kg; Tukey’s HSD, *P* < .05; Fig. 1). Readily available phosphorus (P_sol_) showed large variations without significant differences between plant or MP type (Fig. S2). Lower P availability in planted soils over the course of the incubation was observed as expected. However, plant-available phosphorus (P_available_) did not differ significantly from controls within plant treatments (Fig. 1).

**Figure 2:**
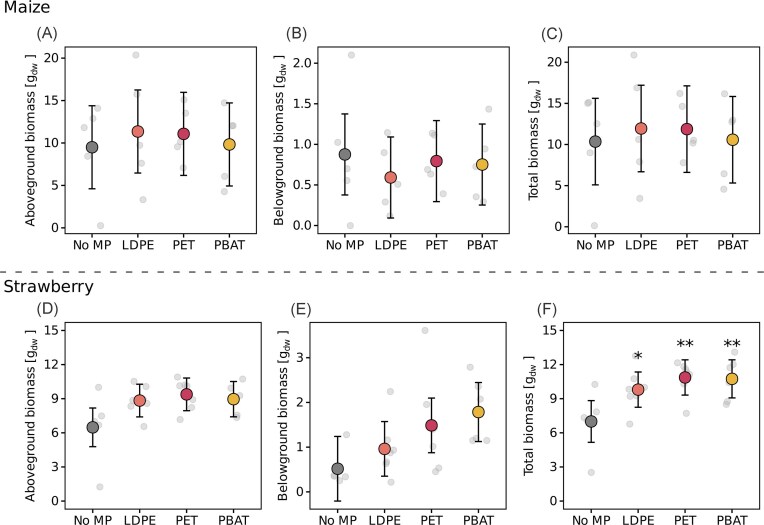
Maize and strawberry biomass differences across treatments (A–F). For aboveground (sum of dry weight of stem, leaves, and fruits), belowground (roots), and total biomass (sum of aboveground and belowground biomass) linear models were fitted separately with MP treatment as the explanatory variable. Estimated marginal means with 95% confidence intervals were calculated from linear models, with underlying raw data depicted in gray. Significant differences between MP treatments and the control (ɑ = 0.05), adjusted for multiple comparisons using the Holm correction, are indicated as asterisks (* for adjusted *P* < .05, ** for adjusted *P* < .01). Comparisons were made with the unamended treatments as the reference group.

MP type explained biologically significant variation in strawberry plant biomass (linear regression models: *R*^2^ = 0.392, *F*(3, 21) = 4.51, *P* = .014; *R*^2^ = 0.284, *F*(3, 21) = 2.78, *P* = .067; *R*^2^ = 0.297, *F*(3, 21) = 2.95, *P* = .056 for total, above- and belowground plant biomass, respectively), but not for maize (Fig. 2, Table S2). Overall, strawberry plant biomass increased under MP treatment, with aboveground biomass rising by 36.3%–44.7% and root biomass by 86%–245%. When combined, total strawberry plant biomass showed a positive correlation with MP treatment in linear models, and estimated marginal means of all MP treatments were statistically significantly increased in MP treatments compared to untreated plants (emmeans, *P* ≤ .02; Fig. 2F, Tables S2 and S3). Our results show that MP treatment increased strawberry plant biomass production, whereas maize remained unaffected. A significant decrease in soil nitrate availability accompanied the increase in strawberry biomass compared to unamended rhizoboxes.

### Soil prokaryotic community-level responses to MP amendment

**Figure 3:**
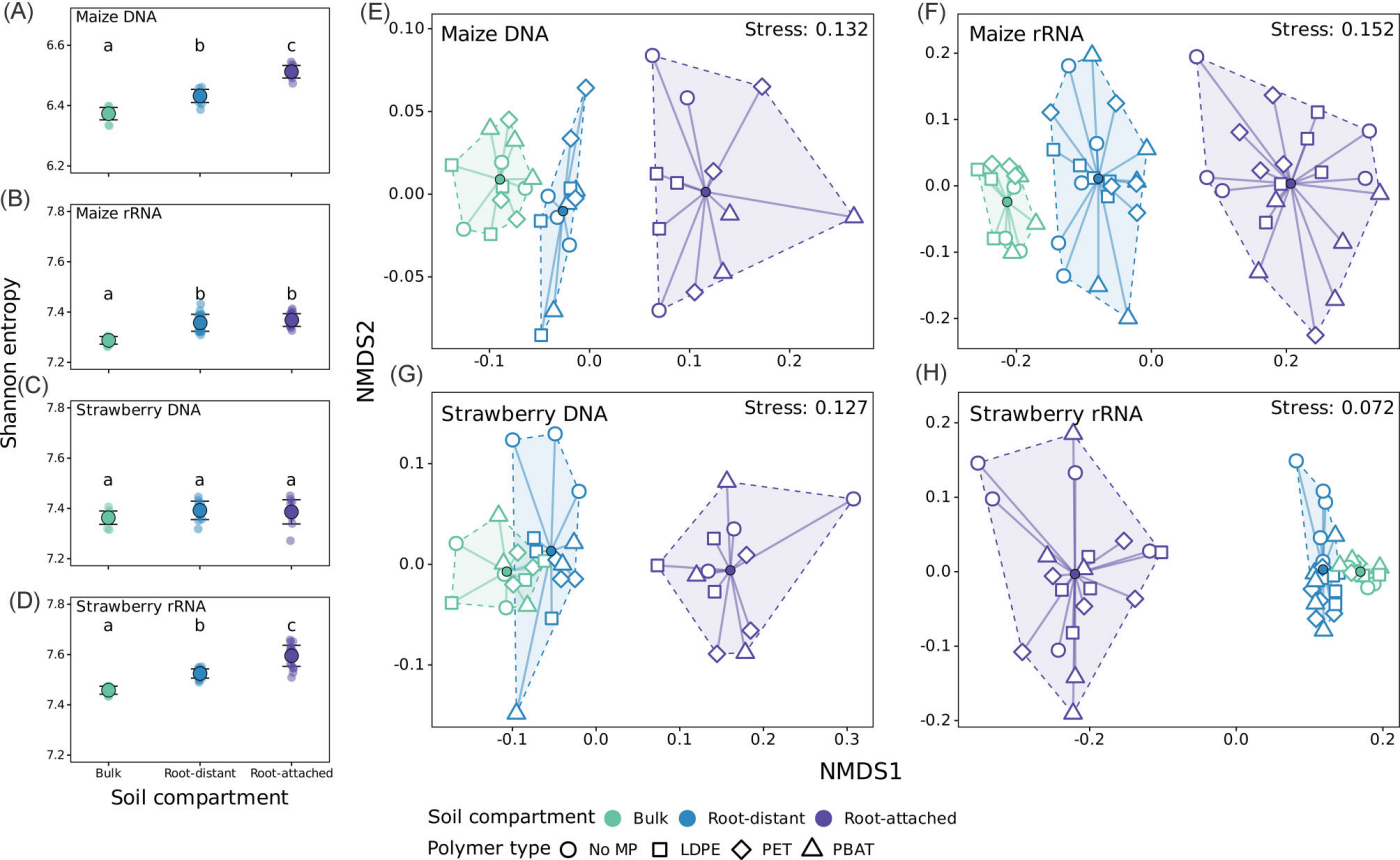
(A–D) Alpha diversity, represented by the Shannon entropy, across different soil compartments. Compact letter displays indicate statistically significant groupings based on Dunn’s *post hoc* comparisons with ɑ = 0.05. (E–H) Bray–Curtis dissimilarities among soil compartments and MP treatments within each library visualized using NMDS ordinations.

Shannon entropy showed significant differences in microbial communities between soil compartments (Kruskal–Wallis followed by Dunn’s *post hoc* test, ɑ = 0.05; Tables S4 and S5). Bulk soil Shannon entropy was significantly lower than that of maize and strawberry rhizosphere soils. This trend was observed for rRNA-derived microbiomes of both plants, and for DNA-profiling of maize (Fig. 3A–D). In contrast, Shannon entropy remained unaffected by MP treatments for all soil compartments (Fig. S3; Kruskal–Wallis test, ɑ = 0.05; Table S4).

The overall structure of DNA and rRNA-derived prokaryotic communities was consistently separated by soil compartments along NMDS axis 1 (Fig. 3E–H). The community composition differed significantly between root-attached, root-distant, and bulk soils (PERMANOVA, pairwise comparisons: all *P* < .01; Tables S6 and S7). Bulk and root-distant strawberry microbiomes exhibited higher similarity to each other than to root-attached communities (Fig. 3E–H). Heterogeneity in group variance across soil compartments was also detected (Table S6), but grouping by soil compartment and respective centroids were clearly distinguished in low-stress NMDS visualizations (Fig. 3E–H). Overall, bulk soil samples showed lower dispersion (tighter clustering) than other soil compartments. Although, DNA and rRNA communities in bulk soil amended with MP did not differ from those in unamended controls, planted soils showed MP-specific community shifts, depending on plant species and soil compartment. Especially in root-distant strawberry soils, rRNA- and DNA-derived communities of MP treatments were clearly separated from the unamended control (Fig. 3G–H).

**Figure 4:**
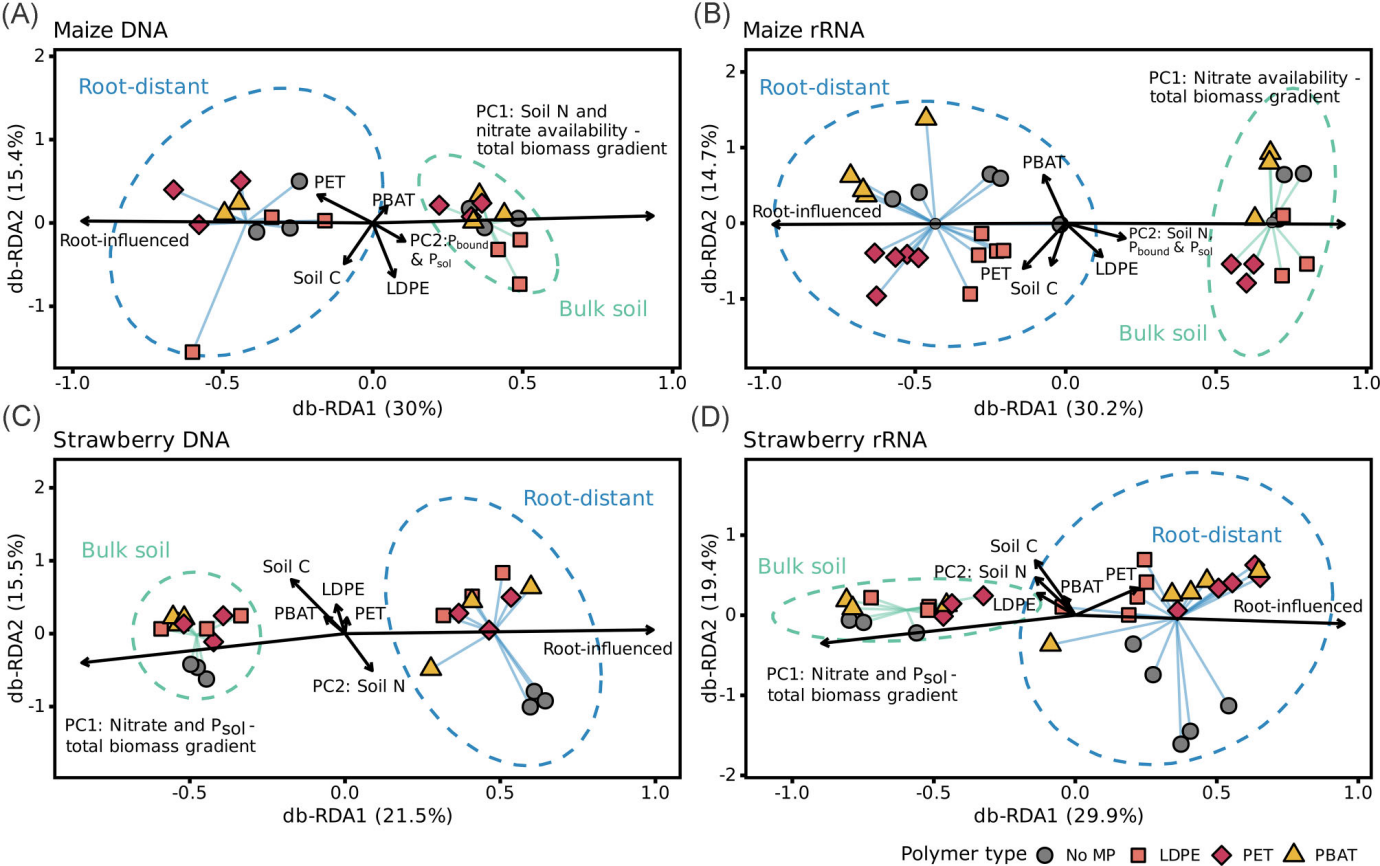
Distance-based redundancy analysis of soil and rhizosphere DNA- and rRNA-derived communities to assess the effects of soil compartment, MP amendment, plant biomass, soil carbon, and nutrients (A-D). Community structure is depicted as Bray–Curtis dissimilarities. The models used the first two axes from a principal component analysis (PC1 and PC2) of plant biomass, soil total N, nitrate, P_bound_, and P_sol_ to account for multicollinearity among plant biomass and soil nutrients. Constrained variance explained: maize DNA = 31.26%, maize rRNA = 23.95%, strawberry DNA = 28.96%, and strawberry rRNA = 27.95%.

The total constrained variance in microbial community composition explained by the experimental predictors (db-RDA) ranged from 27.5% to 37.7% (Fig. 4, Table S9). To account for high collinearity among plant biomass, soil total N, nitrate, P_sol_, and P_bound_, we included the first two principal component axes (PC1 and PC2) from a principal component analysis as predictors in all models, rather than the direct variables. PC1 captured 71%–79% of the variance in the collinear explanatory variables, while PC2 explained an additional 14%–15% (Table S8). In maize DNA and rRNA, PC1 gave most weight to soil nitrate and plant biomass, capturing a gradient from nitrate-rich and no-plant biomass soils to nitrate-depleted and plant-influenced soils (Fig. 4A and B, Table S8). In strawberry DNA and rRNA, PC1 reflected variation in soil nitrate, phosphorus, and plant biomass (Fig. 4C and D). PC2 was weighted mostly by P_sol_ and P_bound_ in maize DNA and rRNA, or by soil nitrogen in strawberry DNA and rRNA (Table S8). Soil compartment consistently accounted for the largest fraction of constrained variance and was the primary statistically significant driver of community structure (ANOVA, *F* = 1.71–3.01, *P* = .001, *R*^2^ = 0.071–0.109; Table S9). Microbial communities in planted soil were further shaped by the gradient represented by PC1, capturing that reduced soil nitrate levels accompanied plant biomass compared to bulk soils. For both strawberry DNA- and rRNA-derived communities, MP treatments clustered closely together in ordination space and were distinct from unamended controls. For strawberry rRNA microbiota, MP treatments, along with PC1, were identified as additional drivers of community composition (PERMANOVA, MP: *F* = 1.43, *P* = .004, *R*^2^ = 0.123; PC1: *F* = 1.33, *P* = .035, *R*^2^ = 0.038; Table S9). This was in line with the NMDS patterns, in which MP-communities clustered separately from no MP-communities, especially in root-distant soil (Figs 3H and 4D). Together, microbial community composition was primarily driven by the soil compartment across all libraries. At the same time, MP treatments had less pronounced, plant- and compartment-specific effects, with rRNA communities showing stronger responses, particularly in strawberry root-distant soils.

### Bacterial taxon-level abundance shifts induced by MPs

**Figure 5:**
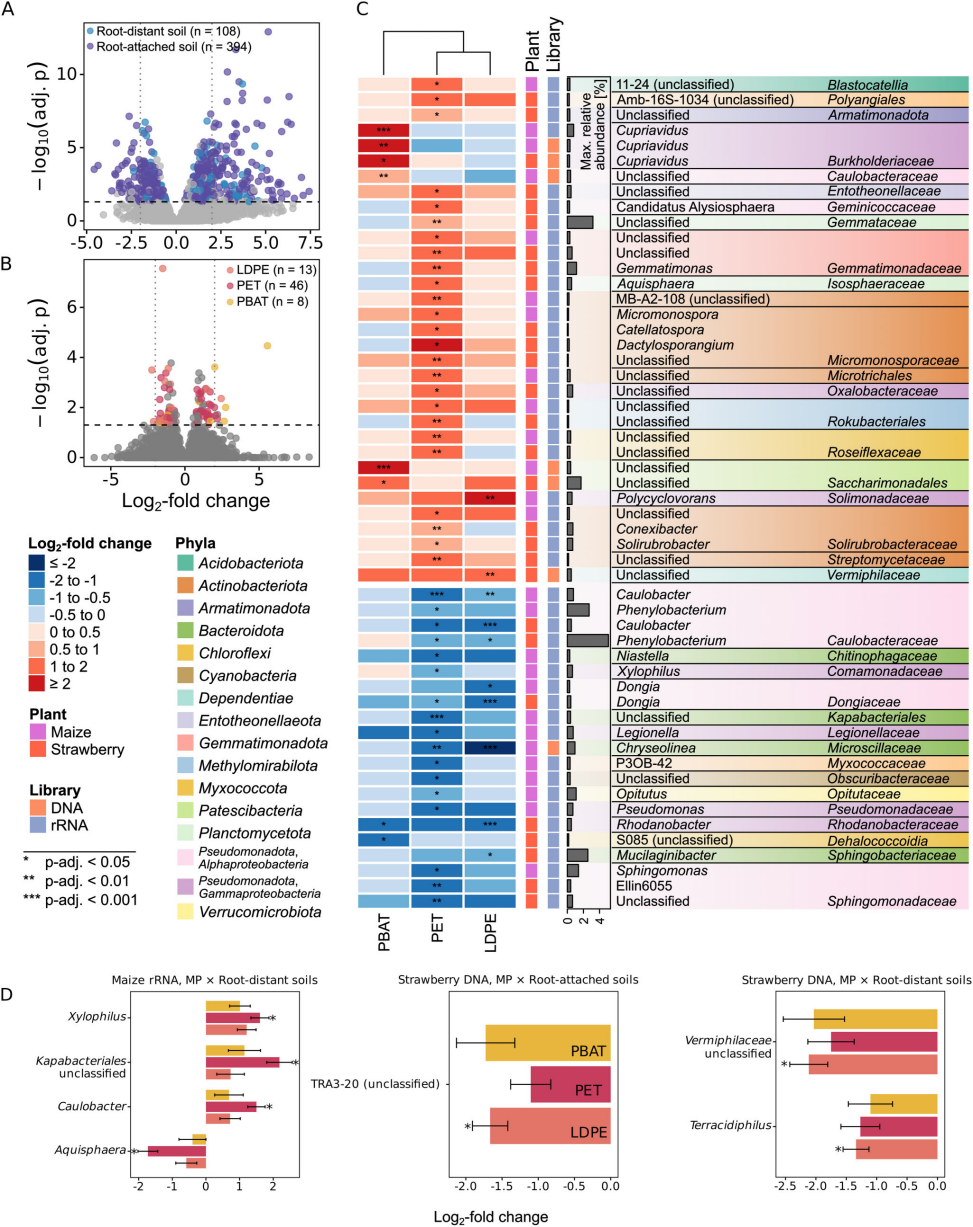
Differential abundance analysis of DNA- and rRNA-derived microbiomes in bulk, maize and strawberry planted soils. (A and B) Log_2_-fold change and adjusted *P*-values from ANCOM-BC analysis using linear models (∼ MP × soil compartment). Taxa with statistically significant differential abundance (ɑ = 0.05, after Benjamini–Hochberg correction) were compared to the respective reference group (A, bulk soil; B, no MP). (C) Log_2_-fold changes of MP-treatments compared to the no MP controls and maximum relative sequencing read abundance of statistically significant MP-affected taxa. (D) Log_2_-fold changes of significant MP × soil compartment interactions.

Differential abundance analyses based on linear regression models were performed to assess taxon-level responses (ASVs aggregated to genus level or lowest resolved taxonomic rank) across root proximities and MP types (modeled as MP × soil compartment). Across all libraries, the impact of soil compartment on microbial community composition was more pronounced than that of MP treatments. In root-distant soils, 108 taxonomic units were differentially abundant compared with bulk soil. At the same time, 394 taxa changed significantly in root-attached soil communities relative to the bulk soil (α = 0.05), with maximum significant absolute log_2_-fold changes of 7.43 (Fig. 5A). At the same time, fewer but still significant taxon-level responses to MP amendments were also identified.

Overall, PET amendment induced the greatest number of changes in taxon abundances (46 taxa). In contrast, only 13 and 8 taxa were differentially abundant in LDPE and PBAT treatments, respectively (max. significant absolute log_2_-fold change of 5.56; Fig. 5B). For the majority of taxa, MP impacts were consistent in direction across soil compartments, with no significant interaction detected. Only for seven taxa, significant interaction effects between MP type and soil compartment were observed (Fig. 5D). Comparing DNA- vs. rRNA-based communities, rRNA libraries showed the highest number of differentially abundant taxa, with only 10 of 61 hits observed in DNA-derived communities (Fig. 5C and D). In both strawberry and maize libraries, the highest number of differentially abundant taxa was identified in PET treatments, with 15 taxa exclusively affected in strawberry rRNA-derived communities and 12 in maize rRNA-derived communities (Fig. S4).

Overall, MP induced shifts in taxon abundance compared to controls were observed across many phyla, independent of soil compartment, with balanced distribution of enriched and depleted taxa for maize and strawberry libraries (Fig. 5C). The maximum abundance of responsive taxa reached up to 5% relative read abundance. The most substantial log_2_-fold change (LFC) was observed for members of the *Saccharimonadales* (LFC = 5.6, *P*-adj. = 3.43 × 10^−5^) in the PBAT treatment of maize-planted soil (DNA library), which were also enriched to a lesser extent in PBAT-treated strawberry soils (LFC = 1.7, *P*-adj. = 0.035). The largest overall negative LFC was observed for *Chryseolinea* spp. in DNA-derived communities of LDPE-treated maize soils (LFC = −2.2, *P*-adj. = 3.17 × 10^−4^). Furthermore, depletion was observed for several *Alphaproteobacteria* (*Caulobacteraceae, Dongiaceae*, and *Sphingomonadaceae*).

For rRNA-derived taxa, members of the *Solirubrobacteraceae* (strawberry: LFC = 0.8, *P*-adj. ≤ 0.04; maize: LFC = 1.6, *P*-adj. = 0.02), *Roseiflexaceae* (strawberry: LFC = 1.1, *P*-adj. = 0.002; maize: LFC = 1.1, *P*-adj. = 0.003), *Gemmatimonadaceae* (strawberry: LFC = 1.1–2.0, *P*-adj. ≤ 0.01; maize: LFC = 1.6, *P*-adj. = 0.048), *Rokubacteriales* (unclassified; strawberry: LFC = 1.4, *P*-adj. = 0.002; maize: LFC = 1.1, *P*-adj. = 0.013), and *Micromonosporaceae* (strawberry: LFC = 1.2–2.1, *P*-adj. ≤ 0.021; maize: LFC = 1.4, *P*-adj. = 0.017) were significantly enriched in PET treatments of both plants. Conversely, *Phenylobacterium* spp. (strawberry: LFC = −0.7, *P*-adj. = 0.026; maize: LFC = −0.7, *P*-adj. = 0.044) and members of the *Sphingomonadaceae* (strawberry: max. LFC = −1.7, *P*-adj. ≤ 0.005; maize: LFC = −1.1, *P*-adj. = 0.013) were significantly depleted. Alterations in taxa in LDPE and PBAT were much lower than in the PET treatments, and no taxon changed consistently across all MP treatments.

Some taxa showed abundance shifts for two MP treatments, i.e. *Caulobacter* spp. was significantly depleted in PET (LFC = −1.2 to −1.3, *P*-adj. ≤ 0.025) and LDPE (LFC = −1.0 to −1.4, *P*-adj. = 0.001) treated soils of both maize and strawberry rRNA-libraries. *Dongia* spp. was significantly depleted in LDPE-exposed strawberry (LFC = −1.1, *P*-adj. = 2.72 × 10^−4^) and maize rRNA-communities (LFC = −1.4, *P*-adj. = 0.024), as well as in PET strawberry rRNA (LFC = −1.0, *P*-adj. = 0.026). *Cupriavidus* spp. was significantly enriched in maize DNA (LFC = 2.7, *P*-adj. = 0.01) and rRNA libraries (LFC = 2.0, *P*-adj. = 2.43 × 10^−4^), as well as in strawberry DNA (LFC = 2.7, *P*-adj. = 0.035) under PBAT. *Rhodanobacter* spp. was significantly depleted in strawberry rRNA under LDPE and PBAT treatments (LFC = −1.5 to −1.7, *P*-adj. ≤ 0.04). Several taxa were depleted in response to LDPE and PET plastics, including *Phenylobacterium* (strawberry rRNA) and *Chryseolinea* spp. (maize DNA).

For several taxa, the MP effect depended on soil compartment, with significant interactions observed for three taxa in strawberry DNA-profiling, and four taxa of maize rRNA (Fig. 5D). Hence, *Kapabacteriales* (unclassified, LFC = 2.2, *P*-adj. = 0.03), *Xylophilus* spp. (LFC = 1.6, *P*-adj. = 0.03), and *Caulobacter* spp. (LFC = 1.5, *P*-adj. = 0.03) were significantly enriched in PET-amended rRNA communities of maize root-distant soil, whereas *Aquisphaera* spp. (LFC = −1.7, *P*-adj. = 0.03) was found depleted. In strawberry root-distant soils treated with LDPE, DNA-derived communities showed depletion of *Terracidiphilus* spp. (LFC = −1.34, *P*-adj. = 0.04) and unclassified *Vermiphilaceae* (LFC = −2.1, *P*-adj. = 0.04). Finally, members of the unclassified TRA3-20 lineage (Edwards et al. 1999) were found to be −1.7-fold depleted (*P*-adj. = 0.03) in strawberry root-attached LDPE-soil DNA-derived communities. Overall, most differentially abundant taxa were primarily associated with soil compartments, while MP effects were largely independent of soil compartments. PET and LDPE were associated with more taxon-level responses than PBAT, and rRNA-derived communities generally showed stronger responses to MP.

## Discussion

Despite extensive research on MP impacts on soil and rhizosphere microbiomes, this study provides an integrated perspective on how conventional (LDPE and PET) and biodegradable (PBAT) MPs can affect total (DNA-derived) and transcriptionally active (rRNA-derived) fractions of soil microbiomes across different root proximities in two plant systems. Our results showed that MP effects on plant biomass and soil nutrient contents differed markedly between strawberry and maize plants (hypothesis I). Soil microbial community composition was primarily influenced by the proximity of the plant roots, with MP amendments contributing to additional differentiation that varied by polymer type and plant species (hypothesis II). At the taxon-level, biodegradable and conventional polymers showed distinct responses, with PBAT associated with more selective changes, while LDPE and PET induced broader compositional shifts (hypothesis III). Finally, community alterations were consistently more pronounced in rRNA-derived profiles than in DNA-based profiles (hypothesis IV). Overall, our findings substantiate that MPs indeed impact soil and rhizosphere microbial communities, particularly apparent for transcriptionally active microbiomes, with potential implications for rhizosphere-associated processes and plant–microbe interactions.

### MP impacts on plant biomass and soil nutrient content depend on plant species

MP pollution has previously been reported to decrease plant growth and photosynthesis marker and to induce plant stress responses (Mészáros et al. 2023, Landi et al. 2025). The observed increase in strawberry biomass alongside decreased plant-available N and P in the investigated soil (Figs 1 and 2) suggested, that MP amendments indirectly enhanced nutrient uptake and promoted plant growth, rather than directly increasing nutrient availability *per se*. As pristine (unweathered) MPs were used in this experiment, direct adsorption of nutrients to plastic surfaces likely was limited, due to their low reactivity, consistent with previous findings (Bartnick et al. 2025). Since this stimulatory effect was observed for both conventional and biodegradable plastics, different drivers may have enhanced strawberry plant growth. Biodegradable PBAT may have promoted microbial activity in the rhizosphere, thereby elevating plant-available nutrient levels, the beneficial effects of conventional MP seem to point toward changes in soil physical properties, such as altered water retention or porosity (Souza Machado et al. 2018, Bakhshaee et al. 2025). Plant traits, such as enhanced rooting and foraging could have fostered strawberry growth, thereby depleting plant-available nitrogen and phosphorus. The generally higher nutrient demands of maize may have masked similar effects in our experiment. Thus, MP-induced changes in soil properties and microbial activity, in combination with plant species-specific traits (e.g. nutrient economies and root architecture), seem to have resulted in contrasting biomass yields and soil nutrient contents. Consequently, MP effects on crop performance cannot be generalized across plant species. This underscores the relevance of considering plant-specific traits when assessing MP impacts within soil–microbe–plant systems.

### MP effects on soil microbiomes were additional to root-driven gradients

Plants strongly influence their surrounding rhizosphere microbiota to promote plant health and growth (Berg and Smalla 2009). In consequence, distinct microbial communities are typically established near plant roots, often with reduced microbial diversity (Trivedi et al. 2020). However, we observed a modest increase in alpha diversity toward the root, which we cannot explain via canonical rhizosphere effects. Possibly, this contradictory pattern may be explained by the spatial constraints of our rhizobox microcosms, or by the fact that diversity responses can vary across different plant species (Fu et al. 2023). Root proximity emerged as the dominant driver of community differentiation (Figs 3E–H and 5A), underscoring the profound influence of plants on microbiome assembly.

MPs have been shown to widely impact soil microbiomes. In particular on plastic surfaces, diversity can be reduced compared to adjacent soil, illustrating the selectivity of the plastisphere (Luo et al. 2022, Rohrbach et al. 2023, Macan et al. 2025). However, overall diversity in MP-treated soils is not necessarily affected by MP presence, as summarized in a recent meta-analysis (Li et al. 2023) and observed also in our study. Nonetheless, MP amendments explained a share of the observed community variance, albeit to a lesser extent than plant presence, suggesting that MP effects were additional to plant root-driven gradients. Moreover, polymer type-specific effects were partially modulated by the plant species. Only in strawberry-planted soils, microbiomes from all MP-treatments were distinct from unamended controls, while for maize, PBAT amendments seemed to activate a microbiome similar to controls. This was in contrast to previous reports of marked shifts in rhizosphere community structure for biodegradable plastics, including PBAT, relative to unamended controls (Liu et al. 2022, 2024, Meng et al. 2023). In line with the discussed differences between maize and strawberry growth in the section above, MP impacts on the community structure may again be context-specific and influenced by plant-specific traits. Overall, our results indicate that MP acts as a secondary factor to root-driven microbiome gradients, which can still influence microbial activities and biogeochemical cycling.

### Different plastic types elicit contrasting taxon-level responses

MP can drive microbial community differentiation in soils and rhizospheres, with microbes colonizing plastic surfaces (the plastisphere microbes) clearly differing from those in the surrounding soil (MacLean et al. 2021, Rohrbach et al. 2023). In our study, MP particles were not separated from the soil before downstream microbiome analysis, although this would have been technically feasible (Jakobs et al. 2023). Thus, most sequencing reads likely represented soil microbes, although MP-associated taxa were not separated and therefore included in the detected communities. Overall, no taxon exhibited consistent abundance changes across all three MP treatments, suggesting that different polymer types exert distinct and specific effects on soil microbiota.

Biodegradable plastics are increasingly promoted as alternatives to conventional plastics. However, their higher bioavailability suggests that they may have a greater influence on soil carbon and nutrient dynamics. In our experiment, two taxa, *Cupriavidus* spp. and the *Saccharimonadales*, responded most notably to the PBAT treatment regardless of plant species and soil compartment. The increased rRNA transcript and gene abundance (for maize DNA only) of *Cupriavidus* spp. suggested an involvement in PBAT degradation. Members of this genus have previously been reported to synthesize intracellular polyhydroxyalkanoates and to possess a broad catabolic repertoire for aromatic compounds, potentially being involved in the degradation of polyesters (Pérez-Pantoja et al. 2008, Mravec et al. 2016, Martínez-Tobón et al. 2018). Soil amendments with biodegradable plastics like PBAT or PLA have notably increased soil respiration over days and weeks (Rauscher et al. 2023, Kim et al. 2025), likely a direct result of stimulated plastic biodegradation (Zumstein et al. 2018). Over the 11–14 weeks of our experiment, we expect at least partial PBAT degradation, whereas it should have been negligible for LDPE and PET (Chamas et al. 2020).

Together with *Cupriavidus* spp., we also found members of the *Saccharimonadales* highly enriched in PBAT-soils, again for both plants and across soil compartments. These still mostly uncultured microbes are members of the Candidate Phyla Radiation and have been reported to be associated with biodegradable and conventional MP in different ecosystems (Rüthi et al. 2020, Weig et al. 2021, Shen et al. 2023). Previous studies have linked members of this group to increased rhizosphere alkaline phosphatase activity under sugar stimulation and to habitats with elevated phosphorus availability (Mason et al. 2021, Wang et al. 2022). In addition, positive correlations between *Saccharimonadales* and nitrogen cycling genes (*nifH* and *amoA*) have been observed in soils exposed to pesticides in a microcosm study (Shi et al. 2021). While none of these correlations are yet proven, *Saccharimonadales* belong to the phylum *Saccharibacteria*. These are described as ultra-small cells, with only a partial aerobic respiratory machinery. Metagenomic reconstructions indicate that they lack many essential biosynthetic pathways, such as amino acid and nucleotide biosynthesis, which compels them to rely on epibiotic, epiparasitic, or ectosymbiotic lifestyles (Nicolas et al. 2021, He et al. 2024). Several isolates from the human oral cavity and wastewater foams have been co-cultured with *Actinobacteria*, suggesting Gram-positives as preferred hosts (Cross et al. 2019, Batinovic et al. 2021, Nie et al. 2022). Moreover, the genome of *Candidatus* Teamsevenus rhizospherense, curated from the oat rhizosphere, indicates close interactions with rhizosphere microbes and plant roots (Starr et al. 2018). Given this background, the enrichment of *Saccharimonadales* in PBAT-amended soils could indicate epibiotic associations with plastic-degrading bacteria, or possibly also fungi. This marked enrichment of yet enigmatic *Saccharimonadales* in PBAT-amended soils calls for a further in-depth elucidation of microbial interactions between taxa potentially involved in plastic degradation and associated commensals, which together shape the environmental impacts of MP pollution.

In contrast to biodegradable PBAT, conventional polymers such as PET and LDPE are considerably much more durable and persistent in soils. Both differ markedly in their molecular structure and their potential interaction with soil microorganisms. LDPE consists of a nonpolar hydrocarbon backbone, making it chemically inert (Chamas et al. 2020). In contrast, PET contains ester groups and aromatic rings, making it more polar and accessible for environmental interactions (Chamas et al. 2020). In line with this, we observed a higher number of differentially abundant taxa (*n* = 46) in PET than in LDPE (*n* = 13), which could be an indirect result of the modulation of soil properties by MP. A substantial number of taxa enriched by PET were typical soil heterotrophs, either reported or assumed to be involved in the utilization of simple and complex carbon compounds. Among these, the *Isosphaeraceae, Gemmataceae*, and *Microtrichales* are known to decompose more complex carbon substrates, such as polysaccharides, cellulose, and complex organic matter (Dedysh and Ivanova 2015, Miksch et al. 2021, Ivanova et al. 2023). Interestingly, *Gemmata obscuriglobus* has been shown to take up proteins in an endocytosis-like manner (Lonhienne et al. 2010, Fuerst and Sagulenko 2014), which allows members of this lineage to access macromolecules via a unique strategy, potentially providing an ecological advantage in PET-amended soils. Several other PET-enriched taxa, like *Polycyclovorans* spp., *Conexibacter* spp., or members of the *Oxalobacteraceae*, are associated with or capable of aromatic hydrocarbon degradation (Gutierrez et al. 2013, Liang et al. 2021). The enrichment of these taxa raises the question of whether plastic-derived compounds could have contributed to these community shifts, even though the plastics used were pristine and rather recalcitrant. Although microbial transformation of MP-derived low-molecular-weight compounds, such as monomers, oligomers, or additives, has been reported (Qiu et al. 2024, Luo et al. 2025), the relevance of such processes in the present experiment remains uncertain.

Compositional changes of most taxa in our study were consistent across soil compartments, while only a few taxa showed specific responses depending on soil compartment. For example, *Caulobacter* spp. were generally depleted in maize and strawberry rRNA communities under PET and LDPE, except for PET-amended maize root-distant soils. The *Caulobacteraceae* family harbors many members with phosphatase gene expression, plant-growth-promoting functions, and catabolic capacities for lignin, cellulose, and hydrocarbons (Abraham et al. 2014, Wilhelm 2018, Luo et al. 2019). Moreover, they have been reported to be potentially involved in the decomposition of biodegradable plastics (Rüthi et al. 2020, Rauscher et al. 2023). Few other PET-enriched taxa, such as *Micromonospora* and *Streptomyces* spp., are also known for plant-beneficial traits. *Micromonospora* spp. can degrade nitrogen-containing organics, while *Streptomyces* can solubilize phosphate (Martínez-Hidalgo et al. 2014, Chouyia et al. 2022). In contrast, a higher number of taxa typically recognized as plant growth-promoting rhizobacteria were depleted by MP. These depletions occurred almost exclusively in PET and LDPE treatments, suggesting that PBAT impacts on soil microbiota were distinct from those of conventional MP. Among those were *Chryseolinea* spp., significantly depleted in LDPE and PET treatments (in maize DNA libraries). This genus is reported to utilize mono- and disaccharides, polysaccharides, and organic acids (Kim et al. 2013). *Sphingomonas* spp., also found depleted, have been associated with plant growth promotion, including the production of phytohormones, the release of siderophores, and antagonistic activity against plant pathogens (Glaeser and Kämpfer 2014). Also, *Rhodanobacter* spp. was depleted, known for plant-beneficial traits, including the production of ammonia and phosphate, the synthesis of indole-3-acetic acid, and the release of siderophores (Woo et al. 2024). Similarly, *Pseudomonas* spp. was depleted, a genus with a vast functional spectrum, ranging from nitrogen fixation to phosphate solubilization (Yan et al. 2008, Miller et al. 2010). However, several genera observed in our study can include both plant-beneficial and -pathogenic members, such as *Pseudomonas* spp. and *Sphingomonas* spp. (Walker et al. 2004, Glaeser and Kämpfer 2014). This clearly complicates the functional interpretation of their contributions in our rhizosphere systems.

Overall, conventional MPs altered community composition more broadly than biodegradable PBAT, as indicated by a higher number of differentially abundant taxa. PET exhibited more pronounced enrichments and depletions than LDPE, which may reflect differences in intrinsic polymer properties and molecular composition that may alter community composition indirectly, e.g. by modulating soil properties (Wang et al. 2025). In contrast, PBAT appeared as a positive selective driver, as previously suggested also for starch-based biodegradable mulching films (Santini et al. 2024), with consistent enrichment of few specific taxa across plant species and soil compartments. This suggests a direct involvement of these taxa in PBAT decomposition, rather than indirect effects. Although functional roles cannot be inferred here, the observed distinct community shifts indicate that conventional and biodegradable MPs differ in their microbiome-induced responses, with potential implications for plant–microbe interactions.

### MP effects were more apparent in rRNA-based community profiling

Several studies have demonstrated that rRNA-derived community profiling, in addition to DNA profiles, can provide more detailed insights into microbial population responses (Ceretto and Weinig 2025, Nash et al. 2025). However, changes in rRNA-derived relative abundances should not be interpreted as a direct measure of population growth, as ribosome concentration does not strictly correlate with cellular growth (Blazewicz et al. 2013). In addition, rRNA-based profiles can be influenced by variations in ribosomal RNA operon copy numbers across and within microbial taxa (Blazewicz et al. 2013). By assessing both DNA- and rRNA-derived community composition, we identified taxa expressing more subtle or dynamic responses to MP pollution, which were not always reflected in DNA-derived communities. In general, changes in soil and rhizosphere community structure were more pronounced in rRNA-derived community profiling. In fact, most of the observed taxon-specific shifts were exclusively detected in rRNA-based community profiles. These shifts would have remained undetected, if impact assessments were only done for DNA. Together, these findings imply that MP impact should best be derived from rRNA-based community profiling in environmental research, particularly when assessing early or subtle microbiome responses.

## Conclusions

MP impacts on microbial communities are context-dependent and emerge from interactions between polymer properties and plant functional traits. In our work, strawberry biomass and decreased soil nitrate content, suggesting altered plant–soil nutrient dynamics and potentially enhanced plant nutrient uptake under MP exposure. Whether such stimulatory effects can also be observed in more complex field settings, when additional factors such as environmental spatiotemporal dynamics, more complex biotic interactions, and varying climate conditions come into play, remains to be tested. Root proximity remained the primary driver of soil microbial community composition, with MPs acting as additional factor. But still, biodegradable and conventional polymers induced contrasting shifts within rhizosphere microbiota, reflecting differences in their intrinsic polymer properties and accessibility to microbes. Biodegradable MP were associated with a more selective response of a few taxa potentially associated with polymer degradation, whereas conventional polymers were linked to changes across a wider range of microbial groups, including taxa commonly associated with plants and biogeochemical functions. These shifts point to polymer-specific influences on rhizosphere functioning, although their functional consequences within plant and soil contexts, particularly under field-relevant settings, require further validation. In this regard, future research may further explore the role of plant-specific traits and growth dynamics in shaping plant–microbe interaction under MP exposure, as well as plastisphere-related processes. Finally, the larger number of MP-impacted differentially abundant taxa detected in transcriptionally active (rRNA-based) communities clearly highlights the merits of rRNA-based profiling in MP impact assessment in soils. All of this adds to the important discussion of the risks and effects associated with MP accumulation in arable soils, and also the ongoing switching from conventional to novel bio-based or biodegradable plastics in agriculture.

## Supplementary Material

fiag040_Supplemental_File
