# Supplementary material for: Conventional and biodegradable microplastics elicit contrasting taxon-level responses in rhizosphere microbiomes of maize and strawberry
Source: FEMS Microbiol Ecol. 2026 Apr 22;102(6):fiag040. doi: 10.1093/femsec/fiag040 (PMC13192445; doi:10.1093/femsec/fiag040)
Supplement: fiag040_Supplemental_File [file fiag040_supplemental_file.pdf]

SUPPLEMENTARY INFORMATION

Conventional and biodegradable microplastics elicit contrasting taxon-level responses in rhizosphere microbiomes of maize and strawberry

Aileen Jung[a,c,*], Ryan Bartnick[b], Daniel Churchill Thomas[a], Eva Lehndorff[b], Tillmann Lueders[a]

[a] *University of Bayreuth, Ecological Microbiology, Bayreuth Center of Ecology and Environmental Research (BayCEER), Dr.-Hans-Frisch-Str. 1-3, 95448 Bayreuth, Germany*

[b] *University of Bayreuth, Soil Ecology, Bayreuth Center of Ecology and Environmental Research (BayCEER), Dr.-Hans-Frisch-Str. 1-3, 95448 Bayreuth, Germany*

[c] *Marine Biological Laboratory, Ecosystems Center and J. Bay Paul Center for Comparative Molecular Biology and Evolution, Woods Hole, MA 02543, USA*

* Correspondence: Marine Biological Laboratory, 7 MBL Street, Woods Hole, MA 02543, USA, E-Mail: ajung@mbl.edu

Table S1: Basic measured parameters of soil investigated.

Parameter	Soil
location (coordinate)	Bindlach, Germany (49.9725°N, 11.6226°E)
classification (WRB)	silt loam (SiL)
sand (%)	22.5
silt (%)	63.7
clay (%)	13.8
pH value	6.5
total C [g kg^{-1}]	15.54
$C_{organic}$ [g kg^{-1}]	15.44
$C_{inorganic}$ [g kg^{-1}]	0.10
total N [g kg^{-1}]	1.57

Table S2: Statistical summary of MP impacts on maize and strawberry biomass. Aboveground (sum of dry weight of stem, leaves, and fruits), belowground (roots), and total biomass (sum of aboveground and belowground biomass). For each biomass compartment, one separate linear model was fitted with MP type as the explanatory variable. R^2 values were calculated from the corresponding linear regression models and represent the proportion of variance explained by MP type. *F*-statistics with degrees of freedom (Df), *P*-value, and omega-squared (ω^2) effect sizes were derived from the corresponding Type II ANOVAs.

Plant	Linear model	R^2	*F*(df)	*P*-value	ω^2
Maize	Aboveground Biomass	0.029	F(3, 16) = 0.16	0.924	0.000
	Belowground Biomass	0.046	F(3, 16) = 0.26	0.856	0.000
	Total Biomass	0.021	F(3, 16) = 0.11	0.951	0.000
Strawberry	Aboveground Biomass	0.284	F(3, 21) = 2.78	0.067	0.176
	Belowground Biomass	0.297	F(3, 21) = 2.95	0.056	0.190
	Total Biomass	0.392	F(3, 21) = 4.5	0.014	0.296

Table S3: Estimated means and associated statistics derived from plant biomass linear models. Statistical comparisons were made using estimated marginal means between each MP treatment and the respective control and reported as Holm-adjusted *P*-values. "NA" indicates no statistical testing was performed due to a non-significant ANOVA for that biomass compartment.

**Plant**	**Linear model**	**MP type**	**Estimated mean [g_{dw}]**	**Standard error**	**Df**	**Lower 95% CI**	**Upper 95% CI**	**Adjusted *P*-value**
Strawberry	Total Biomass	no MP	7.00	0.88	21	5.16	8.83	NA
		LDPE	9.80	0.75	21	8.25	11.35	0.02
		PET	10.87	0.75	21	9.32	12.42	0.01
		PBAT	10.74	0.81	21	9.07	12.42	0.01
Strawberry	Aboveground Biomass	no MP	6.48	0.82	21	4.78	8.18	NA
		LDPE	8.84	0.69	21	7.40	10.27	NA
		PET	9.38	0.69	21	7.94	10.82	NA
		PBAT	8.96	0.75	21	7.41	10.51	NA
Strawberry	Belowground Biomass	no MP	0.52	0.35	21	-0.21	1.24	NA
		LDPE	0.96	0.29	21	0.35	1.57	NA
		PET	1.49	0.29	21	0.88	2.10	NA
		PBAT	1.79	0.32	21	1.13	2.44	NA
Maize	Total Biomass	no MP	10.35	2.48	16	5.09	15.61	NA
		LDPE	11.95	2.48	16	6.69	17.21	NA
		PET	11.87	2.48	16	6.61	17.13	NA
		PBAT	10.58	2.48	16	5.32	15.84	NA
Maize	Aboveground Biomass	no MP	10.35	2.48	16	5.09	15.61	NA
		LDPE	11.95	2.48	16	6.69	17.21	NA
		PET	11.87	2.48	16	6.61	17.13	NA
		PBAT	10.58	2.48	16	5.32	15.84	NA
Maize	Belowground Biomass	no MP	0.88	0.24	16	0.38	1.37	NA
		LDPE	0.59	0.24	16	0.09	1.09	NA
		PET	0.79	0.24	16	0.29	1.29	NA
		PBAT	0.75	0.24	16	0.25	1.25	NA

Table S4: Statistical differences in Shannon entropy among soil compartments and MP treatments within each soil compartment assessed using Kruskal–Wallis tests. The table reports sample size, Kruskal–Wallis H statistic, degrees of freedom, and *P*-value.

Plant	Library	Factor tested	Soil compartment	Sample size	Kruskal–Wallis H	df	*P*-value
Maize	DNA	Soil compartment	NA	36	30.71	2	2.15×10^{-7}
Maize	rRNA	Soil compartment	NA	52	28.13	2	7.79×10^{-7}
Strawberry	DNA	Soil compartment	NA	36	5.38	2	0.068
Strawberry	rRNA	Soil compartment	NA	52	39.75	2	2.33×10^{-9}
Maize	DNA	MP	Bulk soil	12	3.21	3	0.361
Maize	DNA	MP	Root-distant	12	3.62	3	0.306
Maize	DNA	MP	Root-attached	12	3.72	3	0.294
Maize	rRNA	MP	Bulk soil	12	5.36	3	0.147
Maize	rRNA	MP	Root-distant	20	4.14	3	0.246
Maize	rRNA	MP	Root-attached	20	2.39	3	0.495
Strawberry	DNA	MP	Bulk soil	12	3.77	3	0.287
Strawberry	DNA	MP	Root-distant	12	5.67	3	0.129
Strawberry	DNA	MP	Root-attached	12	2.49	3	0.478
Strawberry	rRNA	MP	Bulk soil	12	3.92	3	0.27
Strawberry	rRNA	MP	Root-distant	20	4.49	3	0.214
Strawberry	rRNA	MP	Root-attached	20	4.69	3	0.196

Table S5: Pairwise comparisons of Shannon entropies between soil compartments for each library. Comparisons were conducted using Dunn's *post hoc* test, with *P*-values adjusted for multiple testing using the Benjamini–Hochberg procedure.

Plant	Library	Pairwise comparison	Z scores	*P*-value	Adjusted *P*-value
Maize	DNA	Bulk vs. Root-attached	-5.54	3.01×10^{-8}	9.02×10^{-8}
Maize	DNA	Bulk vs. Root-distant	-2.71	0.0067	0.0067
Maize	DNA	Root-attached vs. Root-distant	2.83	0.0047	0.0070
Maize	rRNA	Bulk vs. Root-attached	-5.12	3.01×10^{-7}	9.02×10^{-7}
Maize	rRNA	Bulk vs. Root-distant	-4.27	1.92×10^{-5}	2.88×10^{-5}
Maize	rRNA	Root-attached vs. Root-distant	0.98	0.3267	0.3267
Strawberry	DNA	Bulk vs. Root-attached	-1.94	0.0527	0.0790
Strawberry	DNA	Bulk vs. Root-distant	-2.07	0.0382	0.1145
Strawberry	DNA	Root-attached vs. Root-distant	-0.14	0.8921	0.8921
Strawberry	rRNA	Bulk vs. Root-attached	-6.23	4.53×10^{-10}	1.36×10^{-9}
Strawberry	rRNA	Bulk vs. Root-distant	-3.16	0.0016	0.0016
Strawberry	rRNA	Root-attached vs. Root-distant	3.55	0.000389	0.000583765

Table S6: PERMANOVA and beta dispersion results based on Bray-Curtis dissimilarities, testing soil compartment and MP treatment effects, and their interaction as explanatory factors.

Plant Library	Factor tested	PERMANOVA					Beta dispersion			
		Df	Sum of Squares	R^2	*F*-value	*P*-value	Sum Sq	Mean Sq	*F*-value	*P*-value
Maize DNA	Soil compartment	2	0.357	0.238	5.219	1.00×10^{-4}	0.006	0.003	6.779	0.003
	MP	3	0.117	0.078	1.144	0.161	4.51×10^{-4}	1.50×10^{-4}	0.131	0.941
	Soil compartment : MP	6	0.202	0.135	0.986	0.486				
	Residual	24	0.820	0.548						
	Total	35	1.497	1.000						
Maize rRNA	Soil compartment	2	0.931	0.153	4.446	1.00×10^{-4}	0.008	0.004	11.076	1.70×10^{-4}
	MP	3	0.351	0.058	1.117	0.121	0.001	1.76×10^{-4}	0.326	0.807
	Soil compartment : MP	6	0.614	0.101	0.977	0.584				
	Residual	40	4.190	0.688						
	Total	51	6.086	1.000						
Strawberry DNA	Soil compartment	2	0.556	0.226	4.861	1.00×10^{-4}	2.51×10^{-4}	1.26×10^{-4}	0.179	0.837
	MP	3	0.192	0.078	1.121	0.188	0.004	0.001	1.409	0.258
	Soil compartment : MP	6	0.339	0.138	0.989	0.487				
	Residual	24	1.373	0.558						
	Total	35	2.460	1.000						
Strawberry rRNA	Soil compartment	2	1.304	0.299	10.757	1.00×10^{-4}	0.022	0.011	22.942	3.9×10^{-8}
	MP	3	0.264	0.061	1.449	0.052	0.004	0.001	0.717	0.547
	Soil compartment : MP	6	0.364	0.083	1.000	0.436				
	Residual	40	2.424	0.557						
	Total	51	4.355	1.000						

Table S7: Pairwise comparisons of Bray-Curtis dissimilarities between soil compartments within each library. PERMANOVA analyses were performed on subsetted libraries to address heterogeneity in group dispersion. *P*-values were adjusted using the Benjamini-Hochberg method to account for multiple testing.

**Comparison**	R^2	***F*-value**	***P*-value**
Maize DNA			
Bulk vs. Root-distant	0.106	2.615	1.00×10^{-4}
Bulk vs. Root-attached	0.260	7.718	1.00×10^{-4}
Root-distant vs. Root-attached	0.178	4.753	1.00×10^{-4}
Maize rRNA			
Bulk vs. Root-distant	0.078	2.530	1.00×10^{-4}
Bulk vs. Root-attached	0.175	6.378	1.00×10^{-4}
Root-distant vs. Root-attached	0.102	4.317	1.00×10^{-4}
Strawberry DNA			
Bulk vs. Root-distant	0.071	1.687	1.00×10^{-4}
Bulk vs. Root-attached	0.243	7.054	1.00×10^{-4}
Root-distant vs. Root-attached	0.204	5.652	1.00×10^{-4}
Strawberry rRNA			
Bulk vs. Root-distant	0.087	2.849	1.00×10^{-4}
Bulk vs. Root-attached	0.321	14.202	1.00×10^{-4}
Root-distant vs. Root-attached	0.251	12.750	1.00×10^{-4}

Table S8: Explained variance and variable contribution of the first two principal component axes (PC1, PC2) from a PCA of soil nutrients and plant biomass.

	Maize DNA		Maize rRNA		Strawberry DNA		Strawberry rRNA	
	PC1	PC2	PC1	PC2	PC1	PC2	PC1	PC2
Soil N	0.151	0.084	0.114	0.239	0.031	0.926	0.096	0.853
Soil nitrate	0.338	0.026	0.312	0.052	0.278	0.004	0.249	0
P_{bound}	0.099	0.402	0.111	0.377	0.239	0.022	0.238	0.062
P_{sol}	0.045	0.439	0.065	0.324	0.269	0.046	0.254	0.085
Total plant biomass	0.367	0.049	0.398	0.008	0.182	0.002	0.162	0
Variance explained (%)	71.5	15.2	71.7	14.9	77	14.2	79.1	14.1

Table S9: Significance of explanatory variables in distance-based redundancy analysis (db-RDA) of microbial community structures. Analyses were based on Bray–Curtis dissimilarities of DNA and rRNA libraries from maize and strawberry soils. Predictors included soil compartment, MP amendment, PC1 and PC2 (first two principal component axes from a PCA of plant biomass and soil nutrients total N, nitrate, P_{sol}, P_{bound}). Significance was tested using permutation-based ANOVA. Degrees of freedom: soil compartment/soil C/PC1/PC2 = 1; MP = 3.

Predictors	Maize DNA			Maize rRNA			Strawberry DNA			Strawberry rRNA		
	F	*P*	R^2	*F*	*P*	R^2	*F*	*P*	R^2	*F*	*P*	R^2
Soil compartment	2.64	0.001	0.109	2.54	0.001	0.08	1.71	0.001	0.071	3.01	0.001	0.087
MP type	1.09	0.135	0.135	1.09	0.083	0.103	1.05	0.083	0.131	1.43	0.004	0.123
Soil C	1.19	0.127	0.049	1.01	0.404	0.032	0.94	0.753	0.039	1.07	0.253	0.031
PC1	1.14	0.136	0.047	1.00	0.421	0.031	1.07	0.268	0.045	1.33	0.035	0.038
PC2	0.90	0.774	0.037	0.92	0.838	0.029	1.11	0.114	0.046	1.06	0.267	0.031
Total R^2	0.377			0.275			0.332			0.31		

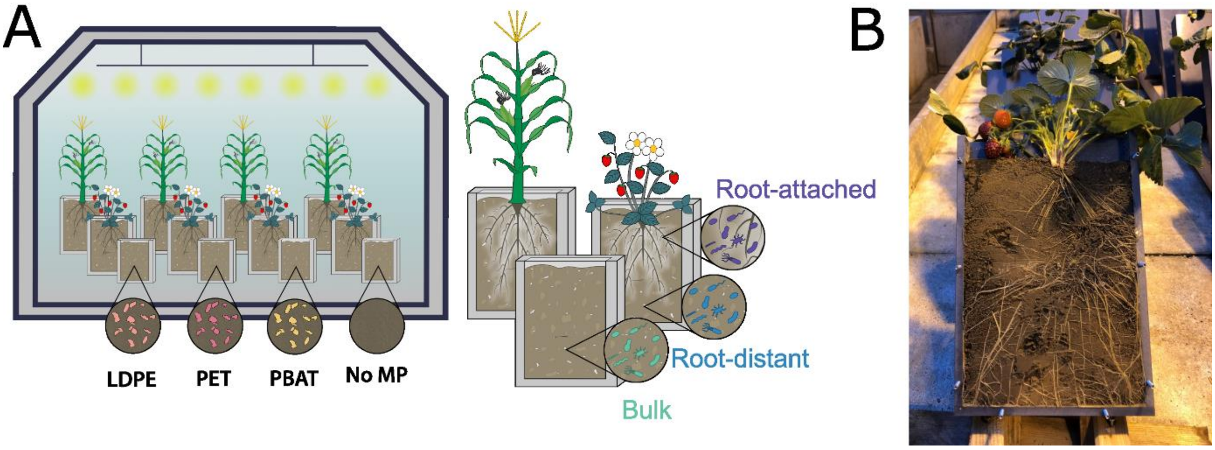

Figure S1: Experimental setup and rhizobox design. A) Schematic illustration of the greenhouse experimental setup with maize and strawberry plants grown in rhizoboxes. At the end of the incubation period, soils were destructively sampled from three compartments: bulk soil, root-distant soil, and root-attached soil. B) Photograph of a rhizobox containing a strawberry plant with visible root growth.

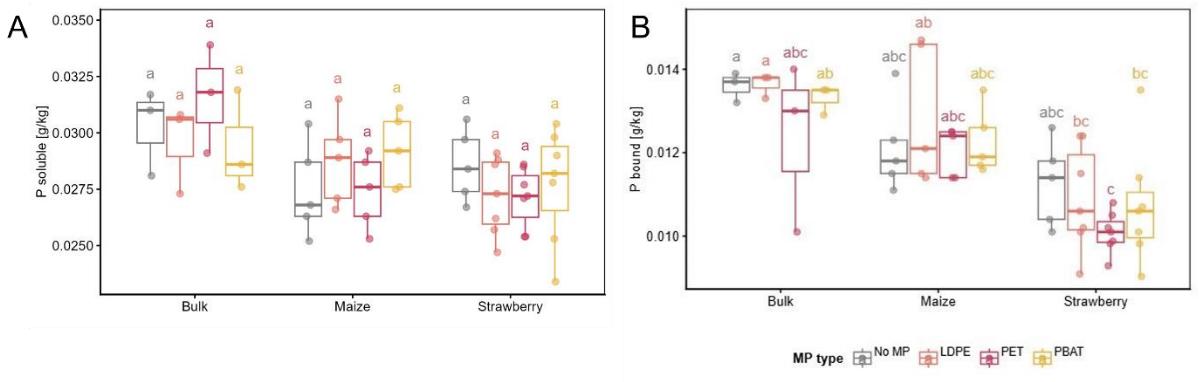

Figure S2: Effects of MP amendments (No MP, LDPE, PET, PBAT) on plant-available phosphorus. A) Readily bioavailable P soluble (P_{sol}), B) plant-available Fe- and Al-bound P (P_{bound}) in unplanted (bulk) and planted (maize and strawberry) soils. Boxplots show median, interquartile range, and outliers; different group letters indicate statistically significant differences among treatments within the same soil environment (bulk, maize-planted, strawberry-planted; Tukey's HSD, $P < 0.05$; same letter = no significant difference).

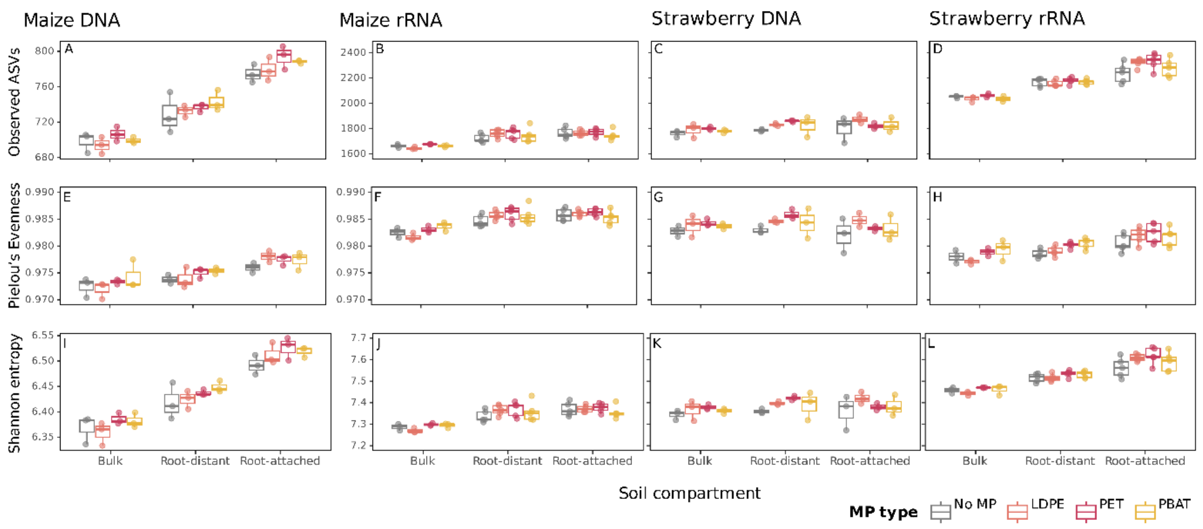

Figure S3: Alpha diversity metrics by soil compartment and MP treatment. Displayed metrics are observed ASVs, Pielou's Evenness (Shannon entropy scaled by richness), and Shannon entropy.

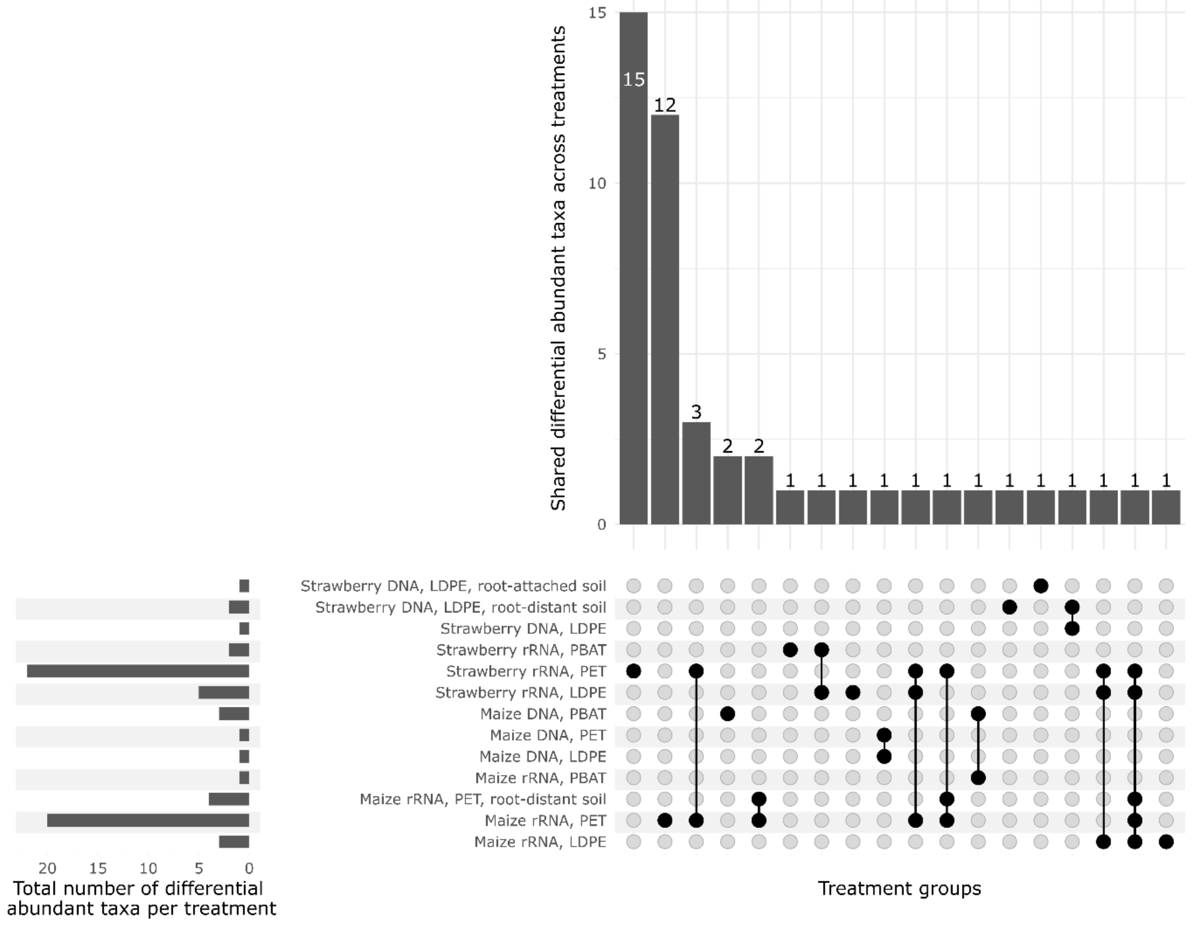

Figure S4: UpSet plot of taxa identified as differentially abundant by ANCOM-BC2 in response to MP. The top panel shows statistically significant taxa unique to, or shared among, treatment groups and libraries. The left panel indicates the total number of statistically significant MP-responsive taxa within each treatment group.
